# β_2_GP1, Anti-β_2_GP1 Antibodies and Platelets: Key Players in the Antiphospholipid Syndrome

**DOI:** 10.3390/antib5020012

**Published:** 2016-05-06

**Authors:** Yik C. Ho, Kiran D. K. Ahuja, Heinrich Körner, Murray J. Adams

**Affiliations:** 1 School of Health Sciences, University of Tasmania, Locked Bag 1322, Launceston, Tasmania 7250, Australia; yik.ho@utas.edu.au (Y.C.H.); kiran.ahuja@utas.edu.au (K.D.K.A.); 2 Menzies Institute for Medical Research, University of Tasmania, Private Bag 23, Hobart, Tasmania 7001, Australia; heinrich.korner@utas.edu.au

**Keywords:** anti-beta 2 glycoprotein 1 antibodies, beta 2 glycoprotein 1, platelet, antiphospholipid antibody, antiphospholipid syndrome, systemic lupus erythematosus

## Abstract

Anti-beta 2 glycoprotein 1 (anti-β_2_GP1) antibodies are commonly found in patients with autoimmune diseases such as the antiphospholipid syndrome (APS) and systemic lupus erythematosus (SLE). Their presence is highly associated with increased risk of vascular thrombosis and/or recurrent pregnancy-related complications. Although they are a subtype of anti-phospholipid (APL) antibody, anti-β_2_GP1 antibodies form complexes with β_2_GP1 before binding to different receptors associated with anionic phospholipids on structures such as platelets and endothelial cells. β_2_GP1 consists of five short consensus repeat termed “sushi” domains. It has three interchangeable conformations with a cryptic epitope at domain 1 within the molecule. Anti-β_2_GP1 antibodies against this cryptic epitope are referred to as ‘type A’ antibodies, and have been suggested to be more strongly associated with both vascular and obstetric complications. In contrast, ‘type B’ antibodies, directed against other domains of β_2_GP1, are more likely to be benign antibodies found in asymptomatic patients and healthy individuals. Although the interactions between anti-β_2_GP1 antibodies, β_2_GP1, and platelets have been investigated, the actual targeted metabolic pathway(s) and/or receptor(s) involved remain to be clearly elucidated. This review will discuss the current understanding of the interaction between anti-β_2_GP1 antibodies and β_2_GP1, with platelet receptors and associated signalling pathways.

## 1. Introduction

Anti-phospholipid (APL) antibodies are a heterogeneous group of autoantibodies targeting different phospholipid binding protein antigens. These autoantibodies include lupus anti-coagulant (LAC), anti-cardiolipin (aCL), anti-beta 2 glycoprotein 1 (anti-β_2_GP1), and anti-prothrombin antibodies [1]. APL antibodies dysregulate normal cellular activities and are associated with recurrent thrombosis (venous, arterial, and microvascular), pregnancy complications (e.g., obstetric failure, pre-eclampsia and eclampsia), and non-specific manifestations (e.g., thrombocytopenia, heart valve disease, chorea, livedo reticularis/racemosa, and nephropathy) [2]. APL antibodies are also present in 1%–5% of healthy populations, including children [3,4]. These populations appear to be asymptomatic, since their autoantibodies are associated with low reactivity [4].

Persistently high levels of APL antibodies, together with specific clinical manifestations, are required for the diagnosis of antiphospholipid syndrome (APS) [1]. APS can occur in isolation or in association with underlying autoimmune diseases such as systemic lupus erythematosus (SLE). The Sydney criteria for the diagnosis of APS recommend that three standard diagnostic assays are used to detect APL antibodies [5]. These diagnostic assays include two enzyme-linked immunosorbent assays (ELISA) that directly detect APL antibodies binding to cardiolipin-β_2_GP1 complexes, or β_2_GP1 only. The third is a clotting assay which indirectly detects APL antibodies by measuring their functional effects on the coagulation system (LAC activity, Table 1) [1,3,6]. Although these assays detect overlapping subpopulations of autoantibodies, their correlation with the clinical manifestations of APS can be varied. LAC assays are superior for detecting pathological subpopulations of APL antibodies when the quality of plasma is maintained [7]. ELISAs for aCL and anti-β_2_GP1 antibodies, however, are weakly associated with thrombotic complications. This may be due to poor standardisation of assays, variable sources and the integrity of β_2_GPI, the secondary calibration process, and/or the assessment and derivation of cut-off values [8]. Consequently, a combination of these tests is used to determine the clinical risk. Patients with persistently high APL antibodies titres (positive in ELISA) and positive LAC activities on at least two occasions, 12 weeks apart, are at higher risk of thrombosis and/or pregnancy complications [1].

The criteria for the diagnosis of APS are well established, yet the interactions between APL antibodies, targeted antigens, and receptors remain unclear. Anti-β_2_GP1 antibodies and their target, β_2_GP1, have become a focus of research for their potential role in thrombosis and pregnancy complications [9]. β_2_GP1-dependent LAC antibodies demonstrate a stronger correlation with thrombosis compared to β_2_GP1-independent LAC antibodies [10,11]. Similarly, β_2_GP1-dependent aCL antibodies are more highly associated with APL antibodies-related complications compared to transient β_2_GP1-independent aCL antibodies induced by infections [12]. Many potential mechanisms of interaction between anti-β_2_GP1 antibodies, β_2_GP1, and cells—e.g., platelets, endothelial cells and monocytes—have been suggested [13]. However, studies investigating the effects of anti-β_2_GP1 antibodies and β_2_GP1 on platelets [14,15,16] may help lead to an improved understanding of their interactions, and consequently, their impact on the haemostatic system [17]. Activation of platelet receptor(s)/metabolic pathway(s) by anti-β_2_GP1 antibodies and β_2_GP1 may result in excessive clot formation and potentially initiate thrombosis and/or pregnancy complications [14,15,16]. Therefore, this review discusses the current understanding of the characteristics and interactions between β_2_GP1 and anti-β_2_GP1 antibodies in relation to platelet receptors and function.

## 2. β_2_GP1

APL antibodies were originally thought to bind directly to phospholipids [26]. In the 1990s, three independent groups demonstrated that APL antibodies actually interacted with phospholipids via β_2_GP1 [27,28,29], significantly raising the interest in this protein. β_2_GP1 had been discovered earlier in 1961 [30], and its amino acid sequence determined in 1984 [31]. It was misnamed apolipoprotein H [32], since it is not an integral part of lipoproteins. Once synthesised in the liver and placenta, β_2_GP1 circulates in blood at a concentration of approximately 4–5 μM. Blood levels of β_2_GP1 are higher in older individuals and in patients with APS, but are lower in pregnant women and patients with stroke and myocardial infarction [33].

β_2_GP1 is an evolutionarily conserved single chain anionic phospholipid-binding glycoprotein, with a molecular weight of approximately 43 kDa [34,35,36]. It belongs to the complement control protein superfamily [37] and consists of 326 amino acids that are arranged in five short consensus repeat, termed “sushi” domains [31,38,39]. The first four domains, each comprising approximately 60 amino acids, are conserved sequences linked together by two disulfide bridges. The fifth domain (DV), however, is a modified form with 82 amino acids. It contains a six residue insertion, a 19-amino acid C-terminal extension and an additional disulfide bond that includes a C-terminal cysteine. These positively charged lysine-rich amino acids (282–287) determine the affinity of β_2_GP1 for anionic phospholipids and negatively charged molecules. DV also adopts a flexible hydrophobic loop (amino acids 311–317), containing a Trp-Lys sequence which is potentially able to insert into membranes. β_2_GP1 has four N-glycosylation sites (Arg143, Arg 164, Arg 174, and Arg 234) located in third domain (DIII) and fourth domain (DIV). There is also one O-linked sugar on Thr130 in β_2_GP1 that accounts for approximately 20% *w/w* of the total molecular mass [40].

### 2.1. Conformations of β_2_GP1

**Figure 1:**
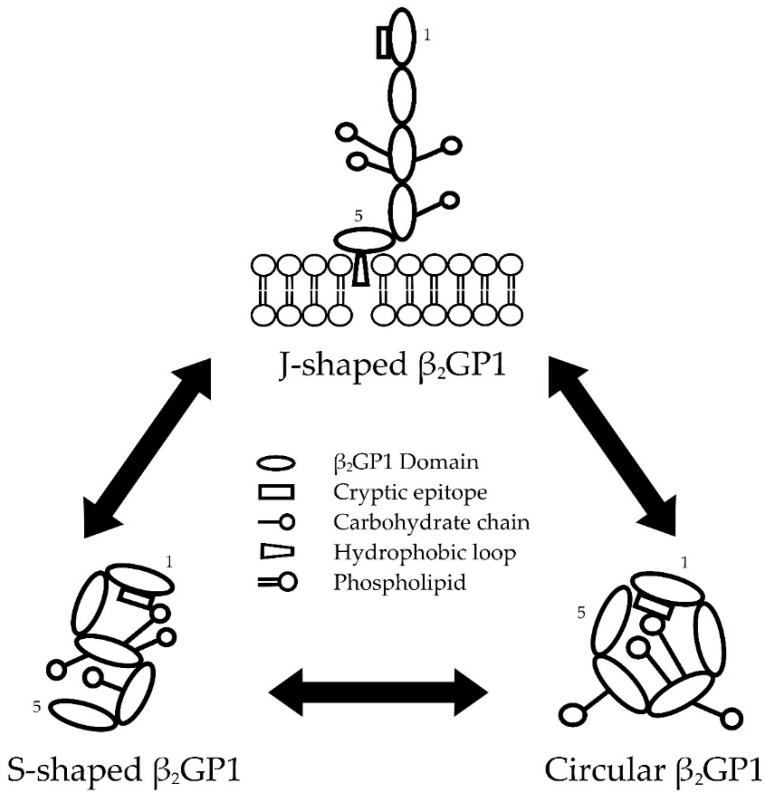
The interchangeable conformations of beta-2-glycoprotein 1 (β_2_GP1). β_2_GP1 is able to transform between three conformations: J-shaped, S-shaped, and circular β_2_GP1. Cryptic epitopes in S-shaped are shielded by carbohydrate chains [43]. Whereas, cryptic epitopes in circular β_2_GP1 are shielded by both carbohydrate chains and domain V [41,44]. Binding of domain V positively charged amino acids and hydrophobic loop to phospholipid membrane breaks the shield on domain I [41]. This exposes the cryptic epitope and allows the binding of clinically significant anti-domain-I-β_2_GP1 antibody.

β_2_GP1 adopts many post-translational modifications which alter the structure and function of the molecule and the exposure of the cryptic epitope [41]. Among them, three interchangeable conformations are more commonly reported (Figure 1). The first conformation was reported by two groups [38,42] based on the crystal structure of the protein. In this conformation, first four domains are stretched with DV at a right angle to the other domains, resembling a J-shape, fish-hook or ‘hockey stick’ conformation. The second reported conformation is S-shaped, as demonstrated using small-angle X-ray scattering [43]. This conformation contains carbohydrate chains from DIII–IV that are twisted and positioned on DI. The third conformation is a common ‘closed’ circular formation present in plasma where DI interacts with DV. This circular formation was initially proposed by Koike *et al.* in 1998 [44], and later directly visualised by Agar *et al.* (2010) using electron microscopy [41].

#### 2.1.1. Transformation between β_2_GP1 Conformations

The discovery of three interchangeable β_2_GP1 structures led to increased understanding of the interaction between anti-β_2_GP1 antibodies and β_2_GP1. These conformational alterations determine the exposure of the cryptic epitope which includes arginine 39–arginine 43 (R39–R43), DI–II interlinker, and possibly aspartic acid residues at positions 8 and 9 [45]. Anti-domain-I-β_2_GP1 (anti-DI-β_2_GP1) antibodies targeting this discontinuous epitope are highly associated with APL antibodies-related clinical manifestations [46,47].

β_2_GP1 is suggested to circulate in an S-shaped or a circular conformation, with less than 0.1% of β_2_GP1 in circulation present in the J-shaped conformation [41,47]. The cryptic epitope in both S-shaped and circular β_2_GP1 is shielded by carbohydrate chains positioned on top of DI [43,48]. In circular β_2_GP1, these negatively-charged carbohydrate chains are also proposed to neutralise the positively-charged DI, allowing the binding of DV [47]. Therefore, S-shaped β_2_GP1 may represent an intermediate form of the molecule as it transforms from a circular to J-shaped conformation [47]. When positively charged amino acids and hydrophobic loop in DV interact with anionic surfaces, β_2_GP1 opens out to the J-shaped conformation, breaking the shield on DI and exposing the cryptic epitope [41].

#### 2.1.2. Factors Affecting β_2_GP1 Conformation

The conformation of β_2_GP1 is dependent on its interaction with anionic surfaces. Its affinity decreases in the presence of ethylene-diamine-tetra-acetic acid (EDTA) [49], and high concentrations of bivalent cations—e.g., calcium and magnesium ions [50]. β_2_GP1 that has been cleaved at DV is also known to have lower affinity [51]. Conversely, dimerisation [52] and increasing β_2_GP1 concentration [50] elevate its affinity. Besides exposure to anionic surfaces, alternations to pH and salt concentration *in vitro* allow structural transformation of β_2_GP1 [41]. High pH and salt concentrations convert circular β_2_GP1 into the J-shaped conformation, and *vice versa* at a low pH and salt concentration. It has also been speculated that these alterations in pH and salt concentration possibly affect the hydrophilic interaction that may be present between DI and DV [41].

APS patients have been proposed to have higher oxidative stress compared to healthy individuals [53]. Oxidative stress favours disulfide bonding between Cys32 and Cys60 (located at DI) and within Cys288 and Cys326 (located at DV) of β_2_GP1. These bonds potentially encourage the binding of anti-β_2_GP1 antibodies to β_2_GP1, and might lead to thrombus formation. Oxidation and biotinylation of β_2_GP1 glycan chains also induce β_2_GP1 dimerisation, which raises β_2_GP1 affinity [54]. Additionally, it is speculated that the intramolecular interaction and conformation of β_2_GP1 can be affected by increased sialylation of β_2_GP1 glycan structures [55].

Lastly, the structure of β_2_GP1 can be inherently diverse. Among the four allelic variants, β_2_GP1 Val/Val genotypes were frequently found to co-exist with anti-β_2_GP1 antibodies [56]. It has also been proposed that the Val247 variant of circular-β_2_GP1 is easier to transform into J-shaped β_2_GP1 after losing the electrostatic interaction between Glu228 (located in DIV) and Lys308 (located in DV) [57]. Thus, this transformation exposes the cryptic epitope for antibody binding and raises the risk of thrombosis.

### 2.2. Physiological Role(s) of β_2_GP1

The precise physiological role of β_2_GP1 is unknown. β_2_GP1-deficient individuals appear to be healthy, suggesting that β_2_GP1 function might not be essential for life [58]. However, the disulphide bonds and phospholipid binding sites in β_2_GP1 are highly conserved across the animal kingdom [36]. Therefore, it is very unlikely that this abundant and well-conserved molecule exists without a function.

Although β_2_GP1-deficient individuals do not have an associated haemostatic abnormality, many functions in the regulation of haemostasis have been attributed to β_2_GP1. First, β_2_GP1 has been demonstrated to inhibit adenosine diphosphate (ADP)-mediated platelet aggregation and serotonin secretion [59,60]. Second, β_2_GP1 might be a mediator for von Willebrand factor (vWF) activation and clearance. β_2_GP1 has been reported to bind to the A1-domain of vWF, preferably vWF in a glycoprotein (GP) Ib-binding conformation. This low affinity binding allows the formation of disulfide bridges between β_2_GP1 and vWF. Thus, the disulfide bridges prevent vWF-mediated platelet activation [15] and potentially protect the cleavage of vWF by the vWF protease, a disintegrin and metalloproteinase with a thrombospondin type 1 motif, member 13 (ADAMTS13) [61]. Thirdly, β_2_GP1 has also been demonstrated to be involved in several coagulation pathways, yet these effects remain to be elucidated [60].

β_2_GP1 has been suggested to be a general scavenger in circulation [62,63]. During apoptosis or cellular activation, the reorganisation of the plasma membrane exposes phosphatidylserine on the cell surface. β_2_GP1 binds to phosphatidylserine expressed on these apoptotic cells [62], as well as platelet microparticles [63], to assist their phagocytosis by macrophages. In addition, β_2_GP1 is also involved in innate immunity as demonstrated by the insertion of DV of β_2_GP1 into bacterial membranes that can lead to cytosol leakage and death of bacteria [64]. β_2_GP1 also changes its conformation while binding to lipopolysaccharide on Gram-negative bacteria, forming a complex which allows recognition and clearance by monocytes [65]. Finally, β_2_GP1 might be important in embryonic development, as the percentage of null offspring born in β_2_GP1 knock-out mice is lower than expected [66].

In summary, β_2_GP1 has been proposed to be involved in a range of physiological processes, including clot formation, fibrinolysis, cell activation, immune responses, atherosclerosis, apoptosis, angiogenesis, and fetal loss [60]. Further research is clearly warranted to determine the precise physiological role(s) of β_2_GP1.

## 3. Anti-β_2_GP1 Antibodies

By itself, β_2_GP1 has no deleterious effect on normal cellular function, but rather interferes with the physiological function of cells following binding with anti-β_2_GP1 antibodies. Therefore, it has been proposed that anti-β_2_GP1 antibodies induce a new function for β_2_GP1 [67]. The affinity of β_2_GP1 is low and only binds to anionic phospholipids below a certain concentration [41,48]. Upon binding with anionic phospholipids, it transforms into the J-shaped conformation and exposes the cryptic epitope located at DI which enables antibodies to bind. When the amount of β_2_GP1 bound to anionic phospholipid membrane reaches a certain density, antibodies dimerise the adjacent β_2_GP1 molecules [48]. This dimerisation forms a high affinity anti-β_2_GP1-β_2_GP1 complex, activating targeted cells and causing APL antibodies-related manifestations.

### 3.1. Clinical Significance of Anti-β_2_GP1 Antibodies

The presence of anti-β_2_GP1 antibodies, especially those with LAC activity, is highly associated with increased thrombotic risk compared to other APL antibody subgroups [10,11]. APS patients have higher levels of platelet activation as reflected by raised urinary thromboxane metabolites [68]. Moreover, the co-existence of J-shaped β_2_GP1 and anti-β_2_GP1 antibodies prolongs the activated partial thromboplastin time of normal plasma, compared to J-shaped β_2_GP1 alone [41], suggesting that anti-β_2_GP1 antibodies also affect secondary haemostasis. Conversely, 40% of APS patients have a prolonged bleeding time without an accompanying bleeding tendency [69]. Although there is no clear explanation for these contradictory findings, it suggests that anti-β_2_GP1 antibodies affect normal haemostatic function.

The contribution of anti-β_2_GP1 antibodies to placental-related pregnancy complications remains controversial. A systematic review and meta-analysis reported that there were insufficient data to support an association between anti-β_2_GP1 antibodies and pregnancy complications [70]. However, an *in vitro* study demonstrated that anti-β_2_GP1 antibodies stimulate trophoblasts to increase secretion of vascular endothelial growth factor, placental growth factor, and soluble endoglin, leading to a higher risk of obstetrical complication [71]. Furthermore, anti-β_2_GP1-β_2_GP1 complexes have been suggested to disrupt the anticoagulant shield formed by annexin A5 on vascular cells [72]. Thus, patients could be predisposed to placental thrombosis that may result in fetal growth restriction and/or pregnancy loss.

### 3.2. Etiology of Anti-β_2_GP1 Antibodies

The etiology of anti-β_2_GP1 antibodies remains unclear. Both genetic and environmental factors may contribute to their production [2,73]. Various animal models and family/population studies have indicated that several human leukocyte antigen genes are associated with the occurrence of APL antibodies and the development of thrombosis [74,75,76]. These pathogenic antibodies are thought to be produced by activated auto-reactive T and B cells due to the similarity between foreign and self-protein/peptide sequences (molecular mimicry) [77]. Viruses, bacteria, mycoplasma and parasites with the same amino acid sequences can also initiate antibody production [78]. However, this theory is unable to clearly explain the etiology, as antibodies are also produced by injecting anionic phospholipids such as cardiolipin, phosphatidylserine, or lipopolysaccharide into animals [79,80].

Anti-β_2_GP1 antibodies might be naturally occurring antibodies, as benign and low affinity APL antibodies are found in 1%–5% of healthy individuals [3,81]. The mechanism(s) of transition of anti-β_2_GP1 antibody from benign to pathogenic are unknown, however there is evidence to suggest that this may be induced by infection. β_2_GP1 binds to pathogenic phospholipids such as protein H from *Streptococcus pyogenes* [82], causing conformational change, exposure of the cryptic epitope, and inducing production of pathogenic anti-DI-β_2_GP1 antibodies. The conformation of β_2_GP1 is also susceptible to many factors and may trigger the synthesis of antibodies. Similarly, antibody production can be prompted by ageing, vaccination, drugs, and malignancies. Their association with clinical manifestations, however, requires further investigation [2,73].

### 3.3. The Two Hit Hypothesis

The detection of anti-β_2_GP1 antibodies in healthy individuals [3,4], APS, and SLE patients without complications [83] indicates that the antibody alone is insufficient for the pathogenesis of APS. It is proposed that a “first-hit” injury primes the endothelium, and a “second-hit” injury triggers thrombus formation. Studies have shown that anti-β_2_GP1 antibodies infused into mice only initiate thrombus formation following vessel-wall injury [84,85]. Endothelium priming involves vessel-wall injury, infection, recent surgery [86], and rarely, the disturbance of redox balance in the vascular milieu [53]. Once primed, the “second-hit” injury, such as smoking, immobilisation, pregnancy, malignancy, *etc.*, stimulates the development of thrombosis [87].

### 3.4. Types of Anti-β_2_GP1 Antibodies

The two hit hypothesis has been proposed to be a good model for the pathogenesis of APS [4]. Yet, it cannot clarify why APL antibodies present in healthy individuals are not pathogenic. Some studies suggest that this could be due to differences in the targeted epitope [10,48] and the structure of anti-β_2_GP1 antibodies [4]. Anti-β_2_GP1 antibodies isolated from primary APS patients are considered to be poly-reactive, as they have been found to react against several domains of β_2_GP1, such as DV (52.9%–64.6%), DIV (45.8%), DI–II (33.1%), and DIII (20.5%) [88]. Anti-DI-β_2_GP1 antibodies recognising the cryptic epitope of DI (Type A) in symptomatic APS patients are strongly associated with thrombotic history and positive LAC activity [10]. Conversely, antibodies that are directed against other domains (Type B) in healthy populations are weakly correlated with thrombosis. These more benign type B antibodies also have lower avidity compared to those pathogenic type A antibodies [89].

Besides binding epitopes, anti-β_2_GP1 antibodies can be classified according to immunoglobulin (Ig) isotype; *i.e.*, IgG, IgM, and IgA. Among these, anti-β_2_GP1 IgG antibodies are more strongly associated with the manifestations of APS [1]. Furthermore, different subclasses of anti-β_2_GP1 IgG antibodies, predominantly IgG2 and IgG3, have also been identified in APS patients and healthy children, respectively [4]. IgG3 is the most effective activator for the classical complement pathway, hence leading to increased C3c (a complement component) activation and binding to anti-β_2_GP1 IgG3 antibodies in healthy children [4]. Complement activation normally triggers platelet activation, which is related to the pathogenesis of APS [90,91]. However, C3c is an opsonin to improve the clearance of the bound target [92]. Instead of activating platelets, C3c binding enhances the clearance of pathogenic anti-β_2_GP1 immune complexes and protects healthy children from complications. Moreover, anti-β_2_GP1 antibodies in healthy and asymptomatic individuals are highly sialylated compared to symptomatic patients [4]. These sialylated anti-β_2_GP1 antibodies have been found to have protective roles for healthy individuals because of their inability to bind and activate platelets.

### 3.5. Anti-DI-β_2_GP1 Antibodies as a Diagnostic Tool

Anti-DI-β_2_GP1 antibodies are highly associated with both vascular and obstetric complications, compared to antibodies against other domains of β_2_GP1 [10]. Anti-DI-β_2_GP1 antibodies are regularly isolated from APS patients compared to those with infection-induced transient APL antibody positivity. APS patients at higher risk of complications (triple APL positivity) also have higher titres of anti-DI-β_2_GP1 antibodies [93], suggesting that the specificity of diagnosis of APS may increase when anti-DI-β_2_GP1 antibodies are included. However, assays that detect anti-DI-β_2_GP1 antibodies have lower sensitivity compared to those that detect the whole β_2_GP1 molecule, as patients might produce clinically significant antibodies against other epitopes [46]. Currently, commercially available kits are not available for the detection of anti-DI-β_2_GP1 antibodies. Instead, research assays with different sensitivities have been reported, such as ELISAs that use N-terminally biotinylated DI on streptavidin plates [94] and a β_2_GP1-DI chemiluminescence immunoassay (CIA, INOVA Diagnostic, San Diego, CA, US) [95]. Further studies are warranted to determine the diagnostic and prognostic value of assays that detect anti-DI-β_2_GP1 antibodies.

## 4. Anti-β_2_GP1-β_2_GP1 Complexes and Platelets

**Figure 2:**
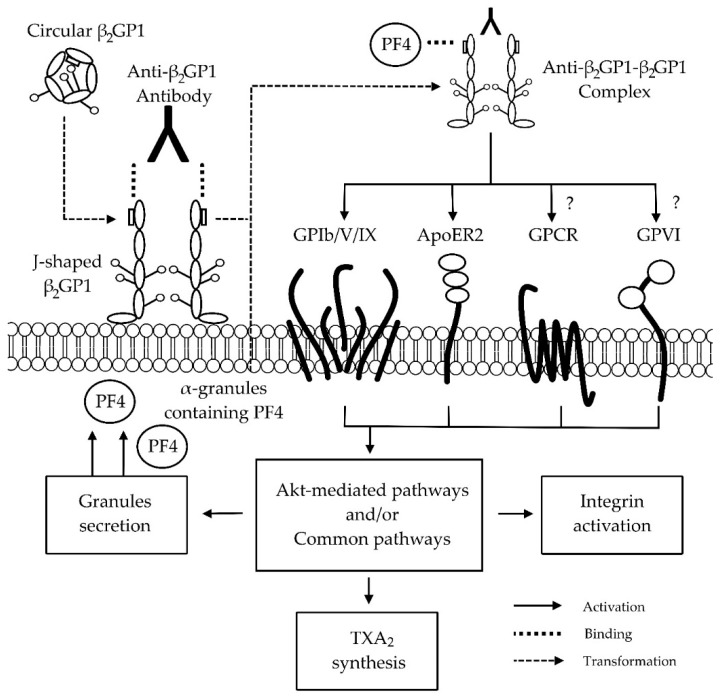
Proposed mechanisms of interaction between anti-beta-2-glycoprotein 1 (β_2_GP1)-β_2_GP1 complex and platelet receptors. Circular β_2_GP1 binds to the anionic phospholipid platelet membrane and transforms into J-shaped β_2_GP1. This allows the anti-domain 1-β_2_GP1 antibody to bind and to form the anti-β_2_GP1-β_2_GP1 complex. The anti-β_2_GP1-β_2_GP1 complex has been proposed to interact with glycoprotein (GP) Ib of GPIb/V/IX [14] and apolipoprotein E receptor 2 (ApoER2) [96,97,98]. In our group, we propose that the complex might trigger adenosine diphosphate (ADP) and collagen-mediated pathways via guanine nucleotide-binding protein coupled receptor (GPCR) and GPVI, respectively [99,100]. Yet, further studies are needed to clarify the variability of results. The binding of the complex with receptors leads to the activation of protein kinase B (Akt)-mediated and/or common pathways, causing granules secretion, thromboxane A_2_ (TXA_2_) synthesis, integrin activation, and subsequently, clot formation. The platelet factor 4 (PF4) from secreted α-granules have also been showed to interact with the anti-β_2_GP1-β_2_GP1 complex [101] Abbreviations: β_2_GP1, beta-2-glycoprotein 1; GP, glycoprotein; ApoER2, apolipoprotein E receptor 2; ADP, adenosine diphosphate; GPCR, guanine nucleotide-binding protein coupled receptor; TXA_2_, thromboxane A_2_; PF4, platelet factor 4; Akt, protein kinase B.

Although there is consensus that β_2_GP1 interacts with anti-β_2_GP1 antibodies to form anti-β_2_GP1-β_2_GP1 complexes with high affinity to anionic phospholipids [41,48], the affected pathway(s) remains unclear. Potential mechanisms by which APL antibodies might increase the risk of vascular and obstetric complications are reviewed elsewhere [13]. In this review, we have only focused on the effects of anti-β_2_GP1 antibodies and β_2_GP1 on platelets (Figure 2).

Platelets are a crucial component of haemostasis, a physiological process that forms a localised clot at the vessel injury site to limit blood loss while maintaining normal blood circulation [17,102]. Activation of platelet receptors leads to platelet adhesion, aggregation, activation of the protein kinase B-mediated and/or common pathways, secretion of granules, integrin activation, synthesis of thromboxane A_2_, and finally, clot formation [17,103]. In the patients with autoimmune diseases, circular β_2_GP1 transforms into the J-shaped conformation after binding to the phospholipid membrane of platelets, allowing anti-β_2_GP1 antibodies to bind and form anti-β_2_GP1-β_2_GP1 complexes [48] (Figure 2). In turn, these complexes are proposed to activate platelet receptor(s)—e.g., glycoprotein (GP) Ib [14], apolipoprotein E receptor 2 (ApoER2) [16], guanine nucleotide-binding protein-coupled receptors-(GPCR) [100], and GPVI [99]. Furthermore, these complexes have also been suggested to affect other pathway(s) by inhibiting β_2_GP1 binding to vWF [15] and by interacting with platelet factor 4 (PF4) secreted from platelets [101]. The activation of platelet receptor(s) by these mechanisms potentially results in excessive clot formation and/or pregnancy complications [14,15,16]. Therefore, understanding the effects of anti-β_2_GP1-β_2_GP1 complexes on platelets is important not only to determine the mechanism(s) of interaction, but to also potentially assist in the development of novel or improved treatments for patients with autoimmune diseases.

It has been reported that β_2_GP1 directly binds to GPIb of the GPIb/V/IX receptor via DII–V [14]. The presence of anti-DI-β_2_GP1 antibodies potentially dimerises β_2_GP1 and inappropriately initiates GPIb-mediated platelet adhesion and aggregation [14,104]. This activation by anti-β_2_GP1-β_2_GP1 complexes may explain the increased thrombotic risk in APS patients [14].

Besides the GPIb receptor, DV of β_2_GP1 has been shown to dimerise and interact with the A1 portion of ApoER2 [96,97,98]. ApoER2, also known as low-density lipoprotein receptor-related protein 8, is the only low-density lipoprotein family receptor found on platelets [96]. This receptor is recognised to be targeted by the anti-β_2_GP1-β_2_GP1 complex, as the blockage of ApoER2 by its antagonist diminishes the effect of the anti-β_2_GP1-β_2_GP1 complex to increase the adhesion of platelets to collagen [105]. It has also been established that the interaction of anti-β_2_GP1-β_2_GP1 complexes with ApoER2 activates platelet analogously to GPIb-mediated platelet activation [16]. Recently, a dimer composed of two A1 portions of ApoER2 joined by a flexible link has been created [98]. This dimer is able to inhibit anti-β_2_GP1-β_2_GP1 complexes from binding to negatively-charged phospholipids and ApoER2 [98], reflecting another possible treatment option for patients with APS.

Anti-β_2_GP1-β_2_GP1 complexes may also affect GPCR and GPVI-mediated platelet activation pathways. Anti-β_2_GP1 antibodies from different origins have recently been reported to exhibit diverse effects on *in vitro* platelet aggregation. Affinity purified rabbit [99] and SLE patient-derived anti-β_2_GP1 antibodies [100] demonstrated inhibitory and enhancement effects, respectively, on ADP-induced platelet aggregation. When collagen was used, affinity purified rabbit anti-β_2_GP1 antibodies [99] enhanced platelet aggregation. However, no effect was demonstrated using patient-derived IgG fractions (containing aCL and anti-β_2_GP1 antibodies) [106] and affinity-purified goat anti-β_2_GP1 antibodies [107]. Based on these results, it is difficult to arrive at a consensus due to the variable effects possibly caused by anti-β_2_GP1 antibodies with different structure and binding specificities. Thus, further research is needed to elucidate the variable effects of anti-β_2_GP1-β_2_GP1 complexes on GPCR- and GPVI-mediated pathways.

As described above, β_2_GP1 binds with vWF to prevent platelet activation. It has been suggested that anti-β_2_GP1 antibodies in APS patients can neutralise this inhibitory effect, potentially leading to thrombosis and consumptive thrombocytopenia [15]. Furthermore, PF4, a pro-coagulant factor secreted from the α granules of platelets, has also been demonstrated to interact with β_2_GP1 [101]. PF4 is proposed to dimerise and stabilise β_2_GP1 on phospholipids, ensuring that β_2_GP1 is easily recognised by anti-β_2_GP1 antibodies. The formation of anti-β_2_GP1-β_2_GP1-PF4 complexes may activate platelets, leading to the development of thrombosis in APS patients [101].

## 5. Conclusion and Further Research

There is substantial literature available on the interaction between three interchangeable β_2_GP1 structures and anti-β_2_GP1 antibodies. The transformation of S-shaped or circular β_2_GP1 to J-shaped β_2_GP1 exposes the cryptic epitope in DI, enabling the binding of anti-β_2_GP1 antibodies, particularly those to DI of β_2_GP1. The formation of the anti-β_2_GP1-β_2_GP1 complex is thought to be responsible for the increased risk of thrombosis and/or pregnancy complications in patients with autoimmune diseases. Although numerous mechanisms of interaction between anti-β_2_GP1-β_2_GP1 complex and receptors/components have been proposed, the actual affected physiological pathway(s) remain unclear. One of the possible explanations for these ambiguities is the use of anti-β_2_GP1 antibodies with different structures and binding specificities from patient- and animal-derived origins across different studies. Therefore, further research is required to better clarify and categorise the type of antibodies used. This approach will in turn facilitate studies that will lead to increased understanding of the interactions between these antibodies and platelets.

In conclusion, the standardisation and development of methods, such as anti-DI-β_2_GP1 antibody ELISAs, are required to differentiate between the types and pathogenicity of anti-β_2_GP1 antibodies. This will allow more meaningful interpretation of laboratory- and clinic-based findings, which will potentially lead to the elucidation of the mechanism(s) of interaction between β_2_GP1, anti-β_2_GP1 antibodies and platelets. In combination, these further developments can help to improve the diagnostic and therapeutic techniques for patients with APS, and perhaps more widely, autoimmune diseases.

## Figures and Tables

**Table 1:** Detection of anti-phospholipid antibodies and their clinical significance.

Assays	Principle of Detection	Antibodies Detected	Clinical Significance [5]
LAC	Clotting assay	LAC (mainly against β_2_GP1 and prothrombin)	Strong correlation with thrombosis [18] and pregnancy morbidity [19].
aCL antibody	Immunological assay	aCL antibody (IgG, IgM, IgA)	Weak correlation with thrombosis and pregnancy morbidity [5,20]. Possible false positive in IgM assay caused by rheumatoid factor or cryoglobulins [21,22]. IgA assay only useful to identify patient subgroups with specific clinical manifestations [5].
Anti-β_2_GP1 antibody	Immunological assay	Anti-β_2_GP1 antibody (IgG, IgM, IgA)	Independent risk factor for thrombosis [23] and pregnancy complications [24]. Higher specificity and lower inter-laboratory variation compared to aCL assay [5]. Clarifies pre-eclampsia and/or eclampsia in pregnant women with negative aCL [24]. Possible false positive in IgM assay caused by rheumatoid factor or cryoglobulins [5]. Presence of IgA might not associate with any clinical manifestation [5].
Anti-prothrombin antibody	Immunological assay	Anti-prothrombin and anti-phosphatidylserine-prothrombin complex	May serve as a confirmatory assay for LAC [25]. Association with thrombotic risk still needs to be clarified [5].

Information collated from Miyakis *et al.* (2006) [5]. Abbreviations: LAC, lupus anti-coagulant; aCL antibody, anti-cardiolipin antibody; Ig, Immunoglobulin; anti-β_2_GP1 antibody, anti-beta 2 glycoprotein 1 antibody.

## Abbreviations

The following abbreviations are used in this manuscript:

APL	Anti-phospholipid
LAC	Lupus anti-coagulant
aCL	Anti-cardiolipin
Anti-β_2_GP1	Anti-beta 2 glycoprotein 1
APS	Antiphospholipid syndrome
SLE	Systemic lupus erythematosus
ELISA	Enzyme-linked immunosorbent assays
D	Domain
Anti-DI-β_2_GP1	Anti-domain I-beta 2 glycoprotein 1
EDTA	Ethylenediaminetetraacetic acid
ADP	Adenosine diphosphate
VWF	Von Willebrand factor
GP	Glycoprotein
ADAMTS13	vWF protease
Ig	Immunoglobulin
ApoER2	Apolipoprotein E receptor 2
GPCR	Guanine nucleotide-binding protein-coupled receptors
PF4	Platelet factor 4
